# Culture Related Factors May Shape Coping During Pandemics

**DOI:** 10.3389/fpsyg.2021.634078

**Published:** 2021-05-19

**Authors:** Ia Shekriladze, Nino Javakhishvili, Nino Chkhaidze

**Affiliations:** Dimitri Uznadze Institute of Psychology, Ilia State University, Tbilisi, Georgia

**Keywords:** COVID-19, anxiety, meaning in life, coping, individualism-collectivism

## Abstract

This study aimed to examine how anxiety related to different styles of coping during the COVID-19 pandemic and how these relationships were moderated by the cultural orientations of individualism/collectivism and a person’s sense of meaning in life. A sample of 849 participants from Georgia completed an online survey during the final stage of lockdown. To measure the main variables, we used the State Anxiety Inventory, the Horizontal and Vertical Individualism and Collectivism Scale, the Meaning of Life Questionnaire, the COVID-19 Worry Scale, and the Ways of Coping Scale tailored to COVID-19 pandemic. The latter measured rational coping via the subscales of information accessing/processing and action-planning coping, and affective coping – via the subscales of passive-submissive and avoidant coping. Results suggested that anxiety positively predicted both affective coping styles and negatively predicted the action-planning coping style, while COVID-19 worry predicted all coping styles; presence of meaning in life positively predicted both rational coping styles and negatively predicted the avoidant coping style, while search for meaning positively predicted all coping styles; individualism negatively predicted the passive-submissive style and positively predicted the action-planning style, whereas collectivism predicted all coping styles; furthermore, individualism and collectivism moderated the link between anxiety and the passive-submissive coping style, presence of meaning in life moderated the link between anxiety and avoidant coping style, while search for meaning in life moderated the link between anxiety and the action-planning coping style. Overall, the findings enrich the cultural transactional theory of stress and coping, and generate insights for the culture-sensitive approach to the meaning in life. The results were conceptualized vis-a-vis Georgia’s intermediate position between clear-cut individualism and clear-cut collectivism.

## Introduction

The COVID-19 pandemic has created a worldwide crisis causing drastic changes and serious stress for populations at large. Although it affects different parts of the world with varying intensity, it nevertheless represents a global threat with an uncertain future course that could potentially leave everyone with a sense of powerlessness and vulnerability. Fortunately, people build resilience in the face of stressful events (Wu et al., 2013; Havnen et al., 2020); however, the degree to which individuals and groups adapt and cope may vary, and it is important to identify the factors to which this variability can be attributed in order to promote a healthy and adaptive response to stress and prevent the global disease from turning into a mental health crisis.

Uncertainty is conceptualized as a source of anxiety (Grupe and Nitschke, 2013), as is the perceived threat to one’s health and well-being (Bar-Haim et al., 2007; Leal et al., 2017). Therefore, increased levels of anxiety during the pandemic (see Bäuerle et al., 2020; Hyland et al., 2020; Huang and Zhao, 2020; Lebel et al., 2020; Lee et al., 2020; Li et al., 2020; Moghanibashi-Mansourieh, 2020; Özdin and Bayrak Özdin, 2020; Roy et al., 2020; Salari et al., 2020; Speth et al., 2020) did not come as a surprise. In line with the multidimensional view of anxiety in literature (Spielberger and Reheiser, 2009; Bäuerle et al., 2020; Wang et al., 2020), we considered it relevant to measure the state anxiety and examine its link with the ways people cope in the context of the COVID-19 pandemic.

To deal with stressful life circumstances, people use a wide range of coping strategies. Lazarus (1993) identified two distinct functions of coping: problem-focused (same as rational) coping, which aims to actively change the stressful environment, and emotion-focused (same as affective) coping, through which a person either alters their own reaction toward the disturbed environment-person relationship or tries to modify the subjective interpretation of it. A study conducted by Leandro and Castillo (2010) found problem-focused coping to be significantly correlated with personal and emotional characteristics typically associated with healthy functioning (e.g., high self-esteem, low anxiety, low depression), while emotion-focused coping showed the reversed associations. Another study (Rahnama et al., 2017) revealed that with increased anxiety, the use of problem-focused coping decreased. Ben-Zur (2009), on the other hand, found problem-focused coping to be positively linked with positive affect and negatively linked with negative affect, while emotion-focused coping positively correlated with both. Moreover, a Polish study on the COVID-19 pandemic linked elevated levels of anxiety with both rational and affective coping (Rogowska et al., 2020). According to Lazarus (1993), both problem-focused and emotion-focused coping could be adaptive at various times, based on the demands of a situation. Hence, our study aimed to examine affective and rational coping that emerged as a response to the pandemic.

Evidence suggests that countries, as cultural units (Schwartz, 2006), are distinguished from one another by their residents’ ways of reacting and coping, with certain cultural features acting as moderating factors to the variability in the adaptiveness of response (Guan et al., 2020). Schwartz (2006, p.138) defines culture as “…the rich complex of meanings, beliefs, practices, symbols, norms, and values prevalent among people in a society.” Studies show that during collective crises people tend to apply behaviors that are within the realms of familiar and already available collective options (Ibanez and Sisodia, 2020; Roy, 2020).

While researchers have proposed a variety of phenomena to explain cultures, individualism and collectivism are the ones most widely examined (Chun et al., 2006). Individualism and collectivism are defined as a set of values, attitudes, and behaviors that prioritize self versus in-group (Triandis et al., 1988; Triandis and Gelfand, 1998). An individualistic orientation focuses on self as a core unit of society, thereby prioritizing individual rights, autonomy, and achievement, whereas a collectivistic orientation considers the group to be the central unit and emphasizes a sense of harmony, a duty to and a coherence with the group, collective norms, and goals (Chun et al., 2006; Shulruf et al., 2007). Thus, people’s self-image in individualist societies typically entails looking after themselves and their immediate families only, while in collectivist societies, they belong to in-groups forming broader self-construal (Hofstede, 2011).

Furthermore, studies show that individualists and collectivists differ in relation to anxiety: Fischer and Boer (2011) conducted a meta-analysis of 123 samples that examined state anxiety in 28 countries and found that a greater level of individualism was connected to less anxiety; however, this effect for state anxiety was reversed at its extreme levels. While uncertainty is recognized as one of the key characteristics of crises prompting higher anxiety among populations, familiarity of response is considered crucial in reducing and containing anxiety (Roy, 2020). Under the circumstances of global catastrophes, culture largely defines what uncertainty is and how it is dealt with and shapes the ways people cope with anxiety.

Individualism and collectivism are also conceptualized as a within-culture personality dimension called idiocentrism versus allocentrism (Triandis et al., 1985; Triandis, 2000) and used interchangeably (Oyserman et al., 2002; Kim et al., 2016; Soenens et al., 2018). It is believed that idiocentrists prevail in individualistic cultures, while allocentrists prevail in collectivist cultures. Yet, members of a culture do not automatically reproduce cultural attributes; rather, these attributes represent fluctuating tendencies, which may or may not be manifested in a particular individual (Singelis et al., 1995). Thus, idiocentrism and allocentrism may vary within a culture and people may vary in terms of endorsement of individualistic/collectivistic values (Oyserman et al., 2002; Soenens et al., 2018). While some consider individual-level individualism/collectivism to be two ends of one dimension, others perceive them as orthogonal or relatively independent constructs that positively correlate with each another (Kim et al., 2016). In the current study we examined culture orientations as personality-level tendencies for individualism/collectivism (Triandis and Gelfand, 1998) with these two dimensions not being mutually exclusive.

In the transactional theory of stress and coping (Lazarus and Folkman, 1984), Lazarus and Folkman attempted to explain stress as a product of the transaction between a person and the complex environment. According to the model, people’s experiences of a stressor vary depending on personal and contextual factors, including capacities, resources, and norms. In their model, the authors differentiated between primary and secondary appraisal. The primary appraisal involves determining whether the stressor poses a threat, whereas the secondary appraisal encompasses an individual’s evaluation of his or her internal and external resources for addressing the threats. Lazarus and Folkman also defined the levels of control. If an individual has resources to handle the stressor, he or she will tend to apply problem-focused coping (primary control); however, if the challenge is overwhelming and beyond one’s capacity to manage, he or she will most likely use emotion-focused coping (secondary control) (Walinga, 2018). Thus, under certain circumstances, affective coping might be more appropriate for promoting adjustment. For instance, in a study on caregivers of individuals with Alzheimer’s disease, the use of fewer emotion-focused strategies predicted higher psychological morbidity (Cooper et al., 2008). We considered the COVID-19 pandemic as a distinct and well-recognized threat, which depending on the local circumstances (e.g., local epidemiological situation, individual well-being and resources) may vary from highly manageable to highly uncontrollable, thereby prompting variations in coping styles. Thus, measuring COVID-19 worry and its links with the anxiety and coping styles was considered highly relevant.

Cultural coping scholars have further elaborated the above model by connecting it with cultural orientations to better explain stress and coping in diverse cultural contexts (Chun et al., 2006; Kuo, 2013). Within the frames of the cultural transactional theory of stress and coping (Chun et al., 2006), both stress and coping are likely to center around the issues of independence for individualists and interdependence for collectivists. Furthermore, the model suggests that individualistic coping is targeted at modifying the external stressor and thus mainly entails problem-focused coping (primary control), while collectivistic coping is inclined toward modifying oneself and therefore tends to apply cognitive avoidance and emotion-focused coping (secondary control). In line with this theory, Lam and Zane (2004) discovered that Asian American students were inclined to respond to interpersonal stress by modifying their thoughts and emotions, whereas other studies on White American students identified their preference of modifying external stressors (Kuo and Gingrich, 2004; Kuo, 2013).

Evidence suggests that under stressful conditions individuals tend to apply their default coping repertoire based on their cultural values. Thus, cultures with a high degree of individualism tend to react in a more independent than interdependent way and are inclined to favor personal freedom over harmony (Markus and Kitayama, 1991; Higgins et al., 2008; Ibanez and Sisodia, 2020). A current study on the pandemic found that tighter, more collectivistic cultures (e.g., East Asian and South Asian cultures) managed to contain the spread of virus more efficiently than looser Western cultures (North America, Western Europe), which was partly attributed to the role of individualism-collectivism (Gelfand et al., 2021). A study on the COVID-19 pandemic from China pinpointed the mental health risks associated with dominant usage of either style of coping, affective or rational, by emphasizing the benefits of diversified coping (Li, 2020). As a within-culture personal difference, in a study conducted in the United Kingdom, an individualistic orientation predicted reduced intention to comply with social distancing requirements, while a collectivistic orientation was linked to the increased intention to comply, and with an overall tendency to exhibit adaptive responses during the pandemic (Biddlestone et al., 2020). Thus, examining the personality dimensions of individualism/collectivism in relation with coping styles was regarded as particularly relevant for our study.

A growing body of research has indicated that people’s reaction to stress (Dymecka et al., 2020; Trzebiński et al., 2020) as well as their ways of coping can be largely defined by meaningfulness in life (e.g., Davis et al., 2000; Halama, 2014; Miao et al., 2017). The study by Schnell and Krampe (2020) showed that crisis of meaning together with COVID-19 stress positively predicted general mental distress among German and Australian participants. Another study conducted in Poland highlighted a buffering role of meaning in life against anxiety, unproductive thinking, and COVID-19 stress (Trzebiński et al., 2020).

In literature meaningfulness is widely viewed in two dimensions called presence of meaning, i.e., one’s subjective appraisal of life as meaningful, and search for meaning, i.e., the process of attainment of meaning (Steger et al., 2006). These are two distinct moderately related constructs (Steger and Kashdan, 2007). There is unequivocal evidence for presence of meaning to be positively linked with a number of aspects of psychological well-being (e.g., Park and Baumeister, 2016; Ostafin and Proulx, 2020). However, research does not provide clear-cut results for search for meaning (e.g., Steger et al., 2009; Grouden and Jose, 2015).

On one hand, evidence (Dezutter et al., 2014) suggested that the presence of meaning in life was central for positive psychosocial functioning, with the most adapted clusters being high-presence - low-search followed by the high-presence – high-search cluster; search for meaning was found to be linked with more maladaptive functioning as the low-presence – low-search cluster was the least adapted cluster, preceded by the low-presence – high-search cluster. On the other hand, the initial generic understanding of these constructs were further elaborated by Steger et al. (2008), who proposed that while presence of meaning would be higher in individualistic societies, collectivistic cultures would be more characterized by the search for meaning – the process which is not expected to result in finding meaning, but, as such, reflects meaningfulness.

Evidence from research on US and Japanese students indeed suggested that American students reported more presence of meaning and Japanese students more search for meaning. In the US, the relationship between presence of meaning and search for meaning was negative, while in Japan the relationship was positive (Steger et al., 2008). In another study, Brassai et al. (2012) discovered that among Eastern European adolescents, presence of meaning and search for meaning strongly correlated with one another, and both showed significant negative associations with behavioral problems as well as significant positive associations with health-enhancing behaviors. Next, Steger and colleagues proposed that the search for meaning in life can be linked with both positive and negative psychosocial functioning and demonstrated that the presence of meaning was strongly associated with life satisfaction when moderated by the search for meaning (Steger et al., 2011). According to Lin and Chan (2020), collectivism can be viewed as a moderator between the search for meaning and well-being. In their study, the search for meaning in life was positively linked with happiness, life satisfaction, and subjective health in collectivist societies, while in societies with lower levels of collectivism, no relations were found between the search for meaning and well-being. These culture-specific findings might be united under an overarching culture-sensitive understanding of meaning in life.

Thus, the generic understanding and the culture-sensitive understanding agree on positive links between the presence of meaning and mental health indicators. However, these perspectives diverge regarding the role of the search for meaning: while the generic approach links it with less favorable mental health outcomes, the culture-sensitive approach regards it more favorable in collectivist cultures (Steger et al., 2008). Hence, meaning in life and its connection with coping in Georgian culture stood out as pertinent objects of interest for our study.

As the COVID-19 pandemic caused multiple abrupt changes worldwide in individuals’ psychosocial realities and quality of life (Jeong et al., 2016; Qiu et al., 2020; Wang et al., 2020), it created a new context in which proper response to and efficient ways of coping with the ongoing stressors acquired critical importance.

### Georgian Socio-Cultural Context and Pandemic

Located in the juncture of Europe and Asia, Georgia is a small lower middle income country (World Bank, 2020) with ancient history and rich cultural heritage and a population of 3.7 million people (GEOSTAT, 2020). The communicability of COVID-19 in Georgia was low by the time of the study. During the period of 3 months between the first identified case on February 26, 2020 and May 25, 2020, the end date of this study, conducted during the final days of quarantine, there were 730 confirmed cases and 12 deaths (World Health Organization, 2020). Nevertheless, people reported experiencing stress due to the ongoing pessimistic news in the media, the lockdown of workplaces, schools, and other public places, the ongoing 9:00 pm curfew that had been enforced since March 31, the elderly members of family to whom the virus presented an acute risk, the lack of social contacts, the associated economic problems, and an unknown future.

Evidence suggests that culture shapes society’s response to a pandemic and influences its prevention strategies at both micro and macro levels (Airhihenbuwa et al., 2020). Prevention strategies put forth by the Georgian Government at the time of our study comprised both individual (person-centered) and collective (people-centered) tiers. At the individual/micro level, people were encouraged to stay at home, wash hands frequently, and wear masks. At the collective-mezo level, people were discouraged to attend large in-person gatherings, arrange funerals or celebrate anniversaries and weddings. All public meetings encompassing more than 10 individuals were banned. At collective–macro level, international travel, inter-city and local public transportation were suspended; schools, universities and offices were moved to distance learning/working, all large-scale events were canceled or postponed. Perhaps the most controversial collective measure taken by the Government was to close down public cemeteries and strongly discourage church gatherings during Easter, which is the most celebrated religious holiday in the predominantly orthodox country of Georgia. Visiting the graves of the deceased family members and loved ones on the Easter holidays is one of the most deeply rooted traditions in Georgia observed by all, irrespective of their religious feelings and identities.

Traditionally believed to be a collectivistic society (Nizharadze, 2001; Surmanidze, 2001; House et al., 2004; Schwartz, 2006), Georgia is characterized by a higher degree of interdependence among its members as manifested by households consisting of several generations and grandparents actively participating in the upbringing of their grandchildren (Tsuladze, 2003). Studies suggest that around 70% of Georgian young adults, including students and married couples, live with their parents/grandparents (Hauschildt et al., 2015; Omanadze et al., 2017). Similarly, the elderly no longer able to take care of themselves are typically cared by their adult children and grandchildren. According to Hofstede Insights Cultural Compass Report (2020), Georgia tends toward a collectivistic culture, characterized by a strong ‘in-group’ society where people feel highly responsible for fellow members of their groups.

Nevertheless, in the context of world cultural clusters, Georgia is believed to be close to the Eastern European cluster (Tkeshelashvili, 2009). Similarly to Eastern European cultures (Gajda and Oie, 2017), Georgian society is becoming more and more Western against the backdrop of globalization. A study of 108 business organizations found individualism largely prevailing (Jamagidze et al., 2011); this is especially true for young working generations that value autonomy (Sumbadze, 2012). Young people nowadays tend to be more independent, financially support themselves, yet it also is typical for them to support their parents and grandparents (Tsuladze, 2003, 2007). Overall, globalization and the rapidly changing socio-cultural environment in Georgia can be considered a transitional backdrop for the growing individualistic trend (Skhirtladze et al., 2016, 2018). A recent study on the impact of the COVID-19 concern on public mental health showed that the worry about loved ones and others getting infected represented the biggest concern for Georgian participants, followed by the uncertainty around the pandemic, concern about income loss, and the restriction of social contacts (Makhashvili et al., 2020). Thus, despite the growing individualism, orientation on others’ wellbeing stood out as a distinct feature in the context of the pandemic.

### The Present Study

Our study examined how cultural and individual characteristics participated in the relationship between anxiety and COVID-19 worry, and various coping styles, namely affective (emotion-focused) and rational (problem-focused) responses to the pandemic. Anxiety and COVID-19 worry were regarded as predictor variables, and problem-focused and emotion-focused ways of coping were considered as outcome variables, whereas cultural orientations and meaning in life were envisaged as moderating variables.

On the basis of existing evidence as well as theoretical knowledge, we hypothesized anxiety to be linked with affective coping; furthermore, we expected COVID-19 worry, as a threat-oriented emotion, to produce stronger links with task-oriented coping. This hypothesis was substantiated by both the transactional theory of stress and coping, which states that when a stressor is manageable people tend to apply rational coping, as well as the general consensus that, as of May 2020, the threat of the pandemic in Georgia was well under control.

Next, consistent with the culture-sensitive approach to meaning in life, we expected the presence of meaning in life to be linked with problem-focused coping, and the search for meaning in life to be associated with both rational and affective styles of coping; we also anticipated meaning in life to moderate the anxiety-coping link so that the presence of meaning in life would weaken the impact of anxiety on coping styles, while the search for meaning in life would enhance it.

Finally, in line with the evidence linking higher levels of individualism with less anxiety as well as the cultural transactional theory of stress and coping, we expected an individualistic orientation would be linked with rational coping, while a collectivistic orientation would accelerate affective coping; in addition, since individualistic and collectivistic self-construals differ, under the circumstances of the pandemic, we assumed individualism would enhance the manageability of the stressor thereby decreasing the associated anxiety, while collectivism would act in the opposite way; therefore, we hypothesized individualism would attenuate anxiety’s effect on coping styles, while collectivism would enhance it. The specific hypotheses are as follows:

Hypothesis 1: Anxiety will positively predict affective styles of coping and negatively predict rational styles of coping, while COVID-19 worry will positively predict rational styles of coping and negatively predict affective styles of coping;

Hypothesis 2: Individualism will negatively predict affective styles of coping and positively predict rational styles of coping, while collectivism will positively predict affective styles of coping and negatively predict rational styles of coping;

Hypothesis 3: Presence of meaning in life will negatively predict affective styles of coping and positively predict rational styles of coping, while search for meaning in life will positively predict both affective and rational styles of coping;

Hypothesis 4: Cultural orientations will moderate the relationship between anxiety and coping styles so that individualism will weaken its effect on affective and rational coping styles, while collectivism will enhance it;

Hypothesis 5: Meaning in life will moderate the relationship between anxiety and coping styles; namely, presence of meaning in life will attenuate the effect of anxiety on affective and rational coping styles, while search for meaning will enhance it for affective coping and will lessen it for rational coping.

## Materials and Methods

### Participants and Procedure

Data were collected via an electronic self-report survey from a convenient sample of 849 participants during the final days of quarantine (May 21–25). The Study’s ethics approval (R/182-20) was obtained from the Ilia State University Ethics Committee. Participants were recruited via social media and other electronic means of communication and were encouraged to distribute the study link among their contacts. To increase participant involvement and reduce sampling bias, a booster was used.

To minimize participant drop-out, the electronic survey link was first piloted and the results were taken into consideration. The link was forwarded with a brief description of the goal of the study and instructions for completion. The potential participants were informed about the anonymity of the survey, the approximate time (15–20 min) needed to complete the questionnaire, and the criteria for participation, which entailed Georgian speaking individuals aged 18 and older.

The study link encompassed several self-report inventories and questions on demographic and socio-cultural variables. Data gathered on participant demographics included information on a variety of individual and household characteristics including age, gender, marital status, education, employment status, and household composition (the numbers of children, elderly, and individuals with chronic illnesses and the total number of household members).

The mean age of the participants was 37.50 (*SD* = 13.37), with the sample consisting of 679 women. Twenty-five percent of the participants lived with three other persons, 32.9% had more than three persons in the households, 28% had an elderly (70+) person in the household, and only 6.60% lived alone. A high number (43%) of the participants were married, 41% were single and 9.3% were divorced; 16% of the participants were students (see Table 1).

**TABLE 1:** Participant demographics.

Age groups	%	Marital status	%	Number of household members	%	Household includes	%	Employment status	%
18–30	39.20	Married	43.00	1 person	6.60	0–5 aged children	22.00	Full-time job	58.50
31–50	44.80	Single	41.20	2 persons	16.50	School-aged children	37.00	Student	16.60
51–70	14.80	Divorced	9.30	3 persons	19.00	Aged 70+	28.70	Self-employed	8.80
71–82	1.20	Widowed	3.30	4 persons	25.10			Unemployed	6.70
		Other	3.20	More than 4 persons	32.90			Part-time job	4.80
								Retired	1.80
								Other	2.70

### Measures

To gather data regarding the variables of interest, we used the State Anxiety Inventory (Spielberger et al., 1983), the Horizontal and Vertical Individualism and Collectivism Scale (Triandis and Gelfand, 1998), and the Meaning in Life Questionnaire (Steger et al., 2006) – all of them internationally recognized as robust measures and previously validated for the Georgian population (Javakhishvili et al., 2016). Two measures – COVID-19 Worry Scale and the Ways of Coping Scale (Gerhold, 2020) – were borrowed from a recent German study (Gerhold, 2020) and, to some extent, were modified. Both measures were tailored to COVID-19 pandemic. The revised German-adapted version of The Ways of Coping Questionnaire (Folkman and Lazarus, 1988) consisted of the Problem-Focused Ways of Coping and Emotion-Focused Ways of Coping subscales.

The State Anxiety Inventory is a 19-item (20 items in the original version) self-report questionnaire which measures a person’s current level of anxiety using a 4-point Likert Scale (e.g., “I feel frightened,” “I am relaxed”). For the sake of consistency with other measurements, a 5-point Likert Scale from fully disagree to fully agree was used. Cronbach’s alpha produced an excellent index (α = 0.93).

The Horizontal and Vertical Individualism and Collectivism Scale is a 16-item self-report inventory with a 5-point Likert Scale from fully disagree to fully agree, which measures an individual’s cultural orientations. Two subscales of horizontal individualism (“I’d rather depend on myself than others”) and vertical collectivism (“Family members should stick together no matter what sacrifices are required”) were maintained after the completion of Confirmatory Factor Analysis (CFA) with the following fit indices: χ^2^ = 69.019, *df* = 12, *p* = 0.00, RMSEA = 0.75, CFI = 0.921, TLI = 0.821, SRMR = 0.043. These two subscales indeed contain items about independence and interdependence. For the individualism sub-scale Cronbach’s alpha amounted to 0.64, for collectivism it equaled 0.65.

The Meaning in Life Questionnaire is a 9-item self-report inventory with a 5-point Likert Scale from fully disagree to fully agree. It measures the extent of a person’s established sense of meaning on one hand and the search for meaning on the other hand (e.g., “my life has a clear sense of purpose”; “I am looking for something that makes my life meaningful,” respectively). Cronbach’s alphas for these two scales were:0.86 and 0.87, respectively.

The Ways of Coping Scale (Georgian version) is an 18-item self-report questionnaire with a 5-point Likert Scale from fully disagree to fully agree. It measures an individual’s problem-focused and emotion-focused coping styles in response to the pandemic. The instrument underwent Confirmatory Factor Analysis (CFA), which yielded satisfactory fit indices: χ^2^ = 393.94, *df* = 127, *p* = 0.01, RMSEA = 0.05, CFI = 0.919, TLI = 0.903, SRMR = 0.053. As a result of CFA, six questions were removed from the original 24-item inventory and four sub-scales were established out of the remaining 18: (1) Action Planning subscale (four items, e.g., “I think carefully about what to do and stick to it”), (2) Information Accessing/Processing subscale (six items, e.g., “I talk to someone who knows about it”), (3) Passive-Submissive subscale (four items, e.g., “It will emerge over time; there is nothing more to do but wait”), and (4) Avoidant subscale (four items, e.g., “I take refuge in daydreams and imagine times when it was better than today”), with the first two constituting problem-focused coping styles, and the last two representing emotion-focused coping styles. Cronbach’s alpha amounted to 0.77 for the action-planning subscale, 0.78 – for information the assessing/processing subscale, 0.68 – for the avoidant subscale, and 0.62 – for the passive-submissive subscale.

The COVID-19 Worry Scale measured concern with COVID-19 using a three-item self-report inventory with a 5-point Likert Scale from fully disagree to fully agree. The scale measured general worry about COVID-19, the fear of being infected by COVID-19, and the fear of a family member getting infected by COVID-19 (“I am worried about COVID-19,” “I fear I might get COVID-19,” “I fear my family member might contract COVID-19”). The first two items were borrowed from a German study (Gerhold, 2020), while the last one was added by us. The scale underwent Exploratory Factor Analysis (EFA) using principle components analysis with Varimax rotation yielding one factor with all three items loading on it. Cronbach’s alpha produced a good index (α = 0.77).

An additional set of questions with a 5-point Likert Scale examined participants’ economic worry, overall outlook on pandemic, and the perceived impact of pandemic on various life domains such as workload, free time, social contacts, psychological state, economic state, as well as its overall impact on one’s life.

### Statistical Analysis

Data were analyzed using the statistical package IBM SPSS version 21.00. Descriptive statistics were calculated and bivariate correlational analyses were performed to explore the links between numerous variables using Pearson’s *r* coefficient. Regression analyses were performed to identify predictors of outcome variables. Finally, moderation models were tested in the PROCESS macro version 3.5. A probability level of 0.05 was used in all statistical tests of significance. Consistency and reliability of the factor loadings were tested by Cronbach’s alpha, with values higher than 0.6 considered appropriate (Taber, 2017).

## Results

### Descriptive Data

Before proceeding with the hypotheses testing, frequencies, mean scores, and standard deviations of the main variables were calculated along with bivariate correlations (see Table 2).

**TABLE 2:** Correlations, means and standard deviations of main variables.

	1	2	3	4	5	6	7	8	9	*M*	*SD*
(1) State Anxiety										2.79	0.76
(2) COVID-19 worry	0.43**									2.77	0.96
(3) Individualism	−0.24**	−0.13**								4.08	0.62
(4) Collectivism	–0.02	0.14**	−0.12**							3.17	0.86
(5) Action-planning	−0.14**	0.17**	0.21**	0.18**						3.52	0.73
(6) Information processing	0.12**	0.36**	–0.03	0.25**	0.50**					3.11	0.76
(7) Passive-submissive	0.31**	0.24**	−0.17**	0.26**	0.04	0.30**				2.70	0.82
(8) Avoidant	0.50**	0.30**	−0.11**	0.16**	–0.05	0.31**	0.56**			2.74	0.88
(9) Presence of meaning in life	−0.26**	–0.01	0.21**	0.13**	0.37**	0.17**	−0.16**	−0.19**		3.59	0.88
(10) Search for meaning in life	0.14**	0.04	0.11**	0.09**	0.08*	0.17**	0.28**	0.23**	−0.14**	3.39	0.94

**p < 0.01; *p < 0.05. For all scales “1” was the minimum and “5” was the maximum.

We found the mean scores of anxiety and COVID-19 worry to be very similar, both amounting to the below average values; moreover, the scores of rational coping styles exceeded the scores of affective coping styles, while individualism markedly surpassed collectivism. The latter difference was corroborated by the analysis of frequencies with 50% of the sample having high individualism scores, whereas only about 18% of the sample producing high collectivism scores.

Correlational analysis showed that age positively correlated with collectivism (*r* = 0.17, *p* < 0.01) and negatively correlated with individualism (*r* = –0.20, *p* < 0.01). In addition, age positively correlated with presence of meaning in life (*r* = 0.08, *p* < 0.05), and negatively correlated with search for meaning in life (*r* = –0.13, *p* < 0.01). Presence and search for meaning in life were in a weak negative correlation with one another (*r* = –0.14, *p* < 0.01), and the same was true for individualism and collectivism (*r* = –0.12, *p* < 0.01). COVID-19 worry strongly correlated with anxiety; it also positively correlated with all styles of coping. Anxiety positively correlated with search for meaning (*r* = 0.14, *p* < 0.01), and negatively correlated with presence of meaning (*r* = –0.26, *p* < 0.01). Significant correlations were established among anxiety and some measures of the perceived impact of the pandemic: namely, anxiety positively correlated with the overall negative impact of the pandemic on one’s life (*r* = 0.43, *p* < 0.01), negative impact on one’s psychological state (*r* = 0.42, *p* < 0.01), and the worry about economic consequences (*r* = 0.26, *p* < 0.01).

Furthermore, the number of participants afraid of contracting COVID-19 appeared quite low (11% – sufficiently or highly afraid) as opposed to the high number of participants worried about the family members contracting the virus (48% – sufficiently or highly worried). A small number of participants (2.60%) reported a history/presence of coronavirus, and even fewer number (0.60%) reported the family history of COVID-19. A majority (72%) of participants expressed worry about the economic consequences, while 66% reported actual or prospective worsening of economic conditions; 69% reported reduced social contacts, while 43% reported worsened psychological state due to the social distancing requirements. In addition, the sample reported slightly reduced job workload (*M* = 2.86, *SD* = 1.33) and somewhat increased free time (*M* = 3.37; *SD* = 1.27) and domestic workload (*M* = 3.54, *SD* = 0.97).

### Hypotheses Testing

#### Predictions

To test the hypotheses and identify predictors of coping styles, we conducted hierarchical regression analysis via entering demographic variables in the first model and psychological variables in the second model. All regression models were statistically significant: *F*(31,817) = 9.00, *p* < 0.01 for information accessing/processing, *F*(31,817) = 10.69, *p* < 0.01 for action planning, *F*(31,817) = 14.68, *p* < 0.001 for avoidant *F*(31,817) = 10.45, *p* < 0.01 for passive-submissive styles of coping. Significant predictors explained 25% of variance (*R*^2^ = 0.25) in the information accessing/processing coping style; 28% of variance (*R*^2^ = 0.29) - in the action planning coping style; 28% of variance (*R*^2^ = 0.28) – in the passive-submissive coping style; and 36% of variance (*R*^2^ = 0.36) – in the avoidant coping style.

Anxiety positively predicted both affective coping styles, and negatively predicted the action planning coping style, while COVID-19 worry positively predicted all coping styles.

Individualism negatively predicted the passive-submissive coping style and positively predicted the action planning coping style, while collectivism positively predicted all coping styles.

Presence of meaning in life positively predicted both rational coping styles and negatively predicted the avoidant coping style, while search for meaning in life positively predicted all coping styles.

The predictors of four coping styles are displayed in Table 3 in the descending order, presenting psychological predictors first, followed by other (e.g., demographic, perceived impact) variables.

**TABLE 3:** Predictors of coping styles.

Predictors of action planning coping style	β	*t*	*p*
Presence of meaning in life	0.30	9.13	0.000
COVID-19 worry	0.20	5.50	0.000
Individualism	0.13	3.78	0.000
State anxiety	–0.12	–2.75	0.006
Search of meaning in life	0.10	3.24	0.001
Collectivism	0.10	3.06	0.002
Positive outlook on pandemic	0.09	2.66	0.008
Worry about economic consequences	0.08	2.34	0.019
Job workload	0.08	2.00	0.046
**Predictors of information accessing/processing coping style**			
COVID-19 worry	0.31	8.53	0.000
Search of meaning in life	0.17	5.41	0.000
Collectivism	0.14	4.16	0.000
Presence of meaning in life	0.14	4.01	0.000
Age	0.11	2.85	0.004
Job workload	0.09	2.23	0.026
Positive outlook on pandemic	0.07	2.00	0.045
Worry about economic consequences	0.07	1.96	0.050
**Predictors of passive-submissive coping style**			
Collectivism	0.24	7.43	0.000
State anxiety	0.22	5.33	0.000
Search of meaning in life	0.21	6.77	0.000
COVID-19 worry	0.15	4.15	0.000
Individualism	–0.09	–2.67	0.008
Job workload	–0.12	–3.10	0.002
Household workload	–0.07	–2.23	0.026
**Predictors of avoidant coping style**			
State anxiety	0.38	9.65	0.000
Search of meaning in life	0.15	4.89	0.000
Collectivism	0.14	4.60	0.000
COVID-19 worry	0.10	2.99	0.003
Presence of meaning in life	–0.08	–2.54	0.011
Perceived negative impact of social distancing on psychological state	0.10	2.92	0.004
Age	0.07	1.98	0.048

*Only significant predictors are shown with standardized regression coefficients, *t*-tests and significance levels.*

Demographic and perceived impact variables also produced valuable predictions: increased job workload, higher economic worry, and optimistic outlook on the pandemic predicted both of the rational coping styles, whereas reduced job and household workload both predicted the passive-submissive coping style; age positively predicted both information accessing/processing and avoidant coping styles; and perceived negative impact of social distancing on psychological state predicted the avoidant coping style.

#### Moderations

Next, we proceeded with moderation analysis in the PROCESS macro version 3.5 (developed for SPSS by Hayes, 2017) which enables mean centering of variables in interaction. While examining the hypothesized models with respect to cultural orientations and meaning in life, we entered all the demographic and perceived impact variables as covariates. The proposed moderating variables were examined both independently and in combination. Only statistically significant interaction models are described below.

According to the results, when examined independently, both individualism and collectivism acted as moderators between anxiety and the passive-submissive coping style.

**FIGURE 1:**
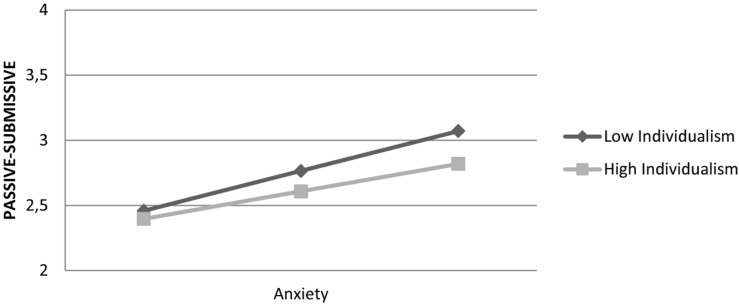
Effect of anxiety on passive-submissive coping style is moderated by individualism.

More specifically, an increase in scores of individualism decreased the effect of anxiety on passive-submissive coping style: interaction was marginally significant, *F*(1,820) = 3.76, β = –0.10, *t*(848) = –1.94, *p* = 0.052. Figure 1 shows that the effect of anxiety on the passive-submissive coping style is stronger at lower levels of individualism. Overall, the model explained 16% of variance in the passive-submissive coping style.

**FIGURE 2:**
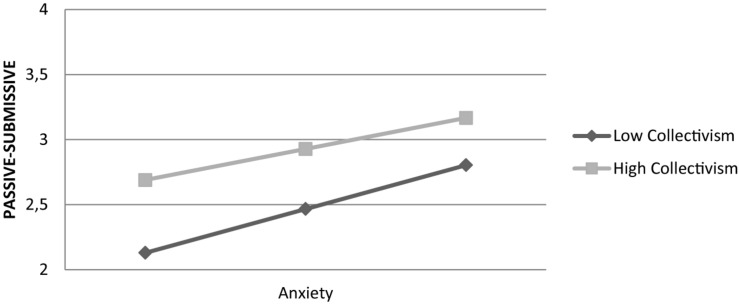
Effect of anxiety on passive-submissive coping style is moderated by collectivism.

An increase in scores of collectivism also reduced the effect of anxiety on the passive-submissive coping style: interaction was significant, *F*(1,820) = 4.27, β = –0.08, *t*(848) = –2.07, *p* = 0.039. Figure 2 shows that the effect of anxiety on the passive-submissive coping style is stronger at lower levels of collectivism. Overall, the model explained 22% of variance in the passive-submissive coping style.

Next, we conducted the above moderation analyses via controlling for collectivism while examining the effect of individualism as a moderator, and vice versa. The moderating effect of individualism was no longer marginally significant (*p* = 0.184), while the effect of collectivism was maintained: β = –0.08, *t*(848) = –2.28, *p* = 0.022.

As far as meaning in life is concerned, when examined independently, both presence of meaning in life and search for meaning in life acted as moderators between anxiety and one of the coping styles.

**FIGURE 3:**
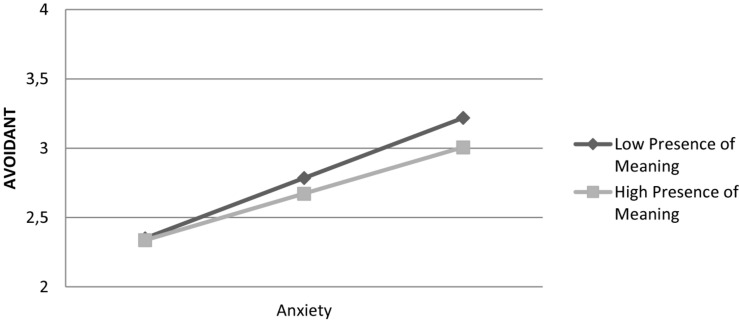
Effect of anxiety on avoidant coping style is moderated by presence of meaning in life.

Presence of meaning in life moderated the relationship between anxiety and the avoidant coping style by attenuating anxiety’s effect: Interaction was significant, *F*(1,820) = 4.39, β = –0.07, *t*(848) = –2.09, *p* = 0.036. Figure 3 shows that the effect of anxiety on the avoidant coping style is stronger at lower levels of presence of meaning in life. Overall, the model explained 31% of variance in the avoidant coping style.

**FIGURE 4:**
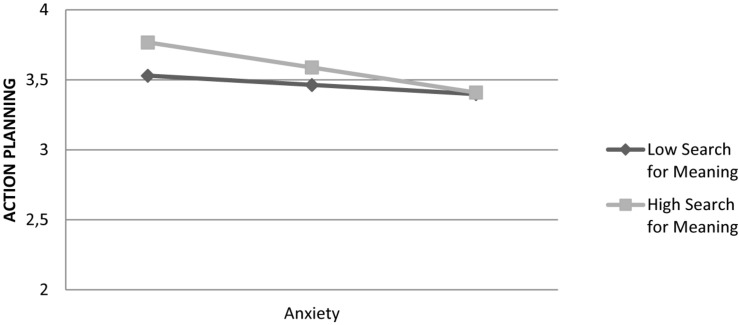
Effect of anxiety on action planning coping style is moderated by search for meaning in life.

Search for meaning in life moderated the relationship between anxiety and the action-planning coping style by attenuating anxiety’s effect: Interaction was significant, *F*(1,820) = 6.71, β = –0.08, *t*(848) = –2.59, *p* = 0.010. Figure 4 shows that an increase in anxiety scores reduced action-planning coping style and this effect was strongest when search for meaning in life was high. Overall, the model explained 15% of variance in the action-planning coping style.

Next, we conducted the above moderation analyses via controlling for search for meaning while examining the effect of presence of meaning as a moderator, and vice versa. The moderating effect of the presence of meaning in life was no longer significant (*p* = 0.070), whereas the effect of search for meaning slightly decreased but was maintained: β = –0.06, *t*(848) = –1.96, *p* = 0.050.

To sum up, when measured independently, cultural orientations exhibited their moderating effect on the relationship between anxiety and passive-submissive coping, whereas the presence of meaning in life impacted the link between anxiety and avoidant coping, and the search for meaning in life impacted the link between anxiety and action planning coping. When controlling for the other variable, individualism lost its moderating power, while collectivism held. Same was true for presence of meaning in life and search for meaning in life: the former could no longer hold the effect, while the latter maintained. However, it is worth noting that for the meaning in life variables, both were still within a similar range of statistical significance (*p*’s = 0.05 and 0.07), and thus the difference in their predictive ability when controlling for the other variable was not as great as that for individualism and collectivism.

## Discussion

Our findings on the relationships between coping and cultural orientations, on one hand, and coping and meaning in life, on the other, in the context of the COVID-19 pandemic, generated a number of insights and thus call for more thorough discussions provided below.

### Anxiety, COVID-19 Worry, Cultural Orientations, and Coping

Our findings revealed that anxiety was highly linked with COVID-19 worry as well as the worry about the economic consequences of the crisis, the perceived negative impact of pandemic on one’s psychological state and on one’s overall life, thereby indicating that the pandemic, as a distinct and immediate threat, indeed represented a major stressor for the sample and its well-being. In line with the study of Makhashvili et al. (2020), the major concern of our participants entailed worrying about the wellbeing of their loved ones.

Furthermore, anxiety positively predicted affective coping and negatively predicted one of the styles of rational coping (action-planning), while COVID-19 worry was linked with all styles of coping. In addition, COVID-19 worry was linked with both collectivism and individualism, thereby confirming the overwhelming nature of the pandemic that more or less equally affected all, from different angles, irrespective of their cultural orientations. Nevertheless, in spite of COVID-19 worry predicting all styles of coping, it still showed stronger links with rational coping thereby confirming its threat-specific nature and its relative manageability. Thus, our findings revealed a subtle difference between state anxiety and COVID-19 worry with respect to coping styles: while COVID-19 worry presented itself as a top predictor of both rational styles of coping, anxiety acted as a top predictor of both affective styles of coping (see Table 3).

Consistent with our findings, other studies have generally found anxiety to be significantly linked with emotion-focused coping and with a decreased use of problem-focused coping (Whatley et al., 1998; Rahnama et al., 2017). In line with our results, studies on the COVID-19 outbreak among Hungarian adults (Szabó et al., 2020) and Chinese adolescents and children (Duan et al., 2020) linked anxiety with increased affective coping and decreased rational coping. Other studies on the COVID-19 pandemic also linked higher levels of anxiety with emotion-focused coping (Mariani et al., 2020; Rogowska et al., 2020), and attributed their stronger link to the uncontrollable nature of the stressor (Mariani et al., 2020). As far as COVID-19 worry is concerned, a study in Germany (Gerhold, 2020) found that, compared to men, women were more inclined to fear COVID-19 and they also used emotion-focused coping in a higher degree; however, no direct links between COVID-19 worry and coping styles were examined.

According to the cultural transactional theory of stress and coping, independence and interdependence are the core values for individualists and collectivists upon which stress and coping are likely to center. In addition, the network of core social contacts of individualists is narrower, consisting of immediate family and friends, while it is broader for collectivists, encompassing extended family, friends, and community (Chun et al., 2006; Kuo, 2013). Thus, threatening one’s autonomy (e.g., extreme restrictions and limitation of freedom) during the pandemic may be particularly stressful for people with an individualistic orientation, whereas threatening interconnections (e.g., social distancing requirements, welfare of others) may pose major risks for people with a collectivistic orientation.

In line with the aforementioned theory, our hypothesis envisaged individualism to be positively linked with rational coping and negatively linked with affective coping, and collectivism – vice versa. Our expectations in regards to individualism were essentially confirmed. However, contrary to our hypothesis, a collectivistic orientation predicted both problem-focused and emotion-focused coping. Thus, our findings indicated that a collectivistic orientation during the global crisis did not necessarily preclude utilizing rational coping but rather widened the coping repertoire. Hence, the question to be addressed below is why, contrary to the proposed theoretical framework, collectivism predicted both styles of coping.

As rightfully pointed out by Lazarus (1993), both ways of coping are appropriate depending on the circumstances, and irrespective of a person’s cultural orientation, task-oriented coping (primary control) is the preferred way of response when one can modify a stressor, while affective coping (secondary control) is more appropriate when an individual has limited/no control over the stressor (e.g., death of a loved one, terminal illness). Examining the pandemic from this angle might be helpful in understanding why collectivistic orientation may accelerate all styles of coping.

More specifically, the cross-cutting enhancing power of collectivism on all styles of coping might be prompted by the circumstances of a pandemic provided that people with such an orientation are worried about their own welfare and the welfare of their family, relatives, and community. This, by no means, implies that people with individualistic orientation are indifferent to community well-being or may not be inclined toward emotional coping; rather, in the context of pandemic, having an individualistic orientation makes one’s circle of concern narrower (e.g., my nuclear family and me) and thus more manageable, for which primary control (i.e., taking precautionary measures for oneself and one’s immediate family) is sufficient. Alternatively, a collectivistic orientation makes one’s circle of concern broader encompassing not only oneself and one’s immediate family but also their extended family, relatives, and friends. As a result, in order to reduce the risks of contracting the virus for oneself and one’s immediate family, a person with collectivistic orientation applies primary control (problem-focused coping), yet ensuring everyone’s well-being (i.e., taking precautionary measures for their extended family, relatives, friends, etc.) is beyond one’s control and, therefore, the increased need for emotional coping (secondary control) arises.

**FIGURE 5:**
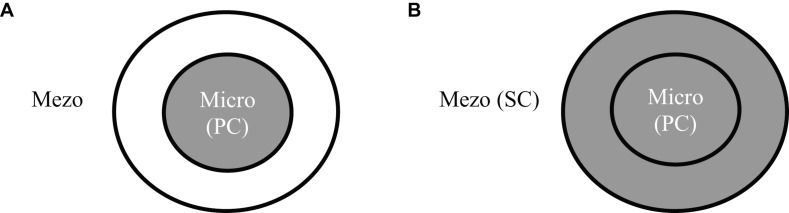
Cultural orientations and coping in the context of pandemic **(A)** Individualism and **(B)** Collectivism. PC, primary control; SC, secondary control.

Thus, the stress of the pandemic, roughly speaking, encompasses micro (a person and his or her immediate family) and mezo (extended family, relatives, and friends) layers for each individual and their cultural orientation has bearing on which layer is activated: in the case of individualism, the micro layer is red-flagged, while in the case of collectivism, both the micro and mezo layers are red-flagged. When only the micro layer is activated, the situation is more manageable, and mainly primary control is used; when both layers are activated, primary control is applied for the micro layer, while secondary control is applied for the mezo layer (Figure 5). Going back to our sample, even though by the time of this study the threat of coronavirus was rather manageable in Georgia, on which basis we hypothesized COVID-19 worry to be linked with rational coping, because Georgians tend to be other-centered (Makhashvili et al., 2020), it prompted the worry about people beyond one’s immediate circle thereby entailing the need for secondary control (affective coping). This may explain why a collectivistic orientation in the context of the pandemic predicted both rational and affective coping.

Consistent with our findings, other studies have also linked collectivistic orientation with both rational and affective coping. A study on British and Japanese students (O’Connor and Shimizu, 2002) showed that Japanese students adopted both emotion-focused and problem-focused coping, while British students favored problem-focused coping. Similarly, in their research on Asian and Caucasian Canadian students, Kuo and Gingrich (2004) examined collective, avoidance, and problem-focused coping and discovered that notwithstanding the participants’ ethnicity, collectivism was positively linked with all three types of coping, while individualism was positively linked with problem-focused coping only.

As a global pandemic is largely beyond one’s control, irrespective of cultural orientations, applying both rational and affective coping may be equally appropriate depending on the local circumstances, and the latter may largely determine the extent to which each is utilized. In fact, a study from Italy demonstrated that collectivistic orientation among young adults predicted lower psychological maladjustment during the COVID-19 pandemic, thereby emphasizing the protective role of goal sharing, interdependence, and sociability (Germani et al., 2020). In another study from Turkey, uncertainty intolerance, typically higher in the case of individualistic orientation and lower in the case of collectivistic orientation, was linked with increased fear of COVID-19 and lower psychological well-being (Satici et al., 2020).

Furthermore, our findings from moderation analyses indicated that both individualism and collectivism may reduce anxiety’s effect on coping. Interestingly, the results suggested that people with high collectivism exhibited markedly higher use of passive-submissive coping without experiencing anxiety compared to people with low collectivism; when anxiety rose though, its boosting effect on passive-submissive coping increased in both cases, being more notable in the case of low collectivism. In line with the proposed transactional person-environment-culture-coping framework, in societies with higher collectivism, both stress and coping are centered on interdependence (Chun et al., 2006). As a result, on one hand, a broader self-construal may generate increased worry about the wellbeing of in-groups originating higher need for affective coping; yet, on the other hand, the broader self-construal may also offer an extended network of support. Hence, higher usage of passive-submissive coping in the absence of anxiety can be attributed to collectivists’ broader circle of concern and the associated need for secondary control. The same broader circle of concern, i.e., the stronger informal support system that such individuals tend to enjoy, may also explain collectivism’s role in weakening anxiety’s effect on passive-submissive coping. Although the support system *per se* was not examined in our study, our results showed that the household composition of most participants included several individuals (32% lived with more than three people). Alternatively, these findings may insinuate that collectivism is so strongly associated with the passive-submissive style that anxiety can no longer make a difference.

Thus, our findings expanded and enriched the cultural transactional theory of stress and coping, linking collectivism with both primary and secondary control. Moderation analyses also suggested that after all individualism and collectivism do not represent two ends of one dimension. They also informed on how complex environments may shape coping with a global stressor in light of cultural orientations.

### Meaning in Life and Coping

As discussed earlier, the role of the search for meaning in life with respect to psychological well-being is not straightforward: while the generic approach states that the search for meaning in life positively predicts mental health problems, the culture-sensitive approach suggests that in collectivist societies it positively predicts mental well-being. These approaches have not been tested on coping styles, thus, our study adds value to the theory. Consistent with the culture-sensitive perspective, we hypothesized the search for meaning in life to positively predict both coping styles.

First of all, in line with a more generic approach, our results showed that anxiety negatively correlated with the presence of meaning in life and positively correlated with the search for meaning in life (see Table 2), thereby confirming the advantage of the presence of meaning with respect to mental well-being. Nevertheless, our results from the regression and moderation analyses corroborated the culture-sensitive approach: despite our sample showing multiple individualistic tendencies, the search for meaning predicted both affective and rational coping, and attenuated anxiety’s negative effect on the action planning coping style.

Furthermore, Dezutter et al. (2014) identified that the combination of high presence and low search showed the strongest link with positive psychological functioning, while the combination of low presence and low search produced the poorest link. These findings demonstrated a certain protective role of the search for meaning even among individualistic societies: when meaning in life is absent, engaging in search for meaning is more favorable than not striving to acquire meaning at all. Hence, a protective role of the search for meaning evident to a certain degree even in the individualistic societies might be more prominent in cultures with higher collectivism. Thus, the mixed nature of our findings with respect to meaning in life partly confirming the generic approach and partly confirming the culture-sensitive approach might be attributed to Georgia’s intermediate and ever-evolving position between pure individualism and pure collectivism.

### Georgian Socio-Cultural Context and Coping

The study findings yielded interesting insights for Georgian culture that can be applied to similar cases. The descriptive statistics of our sample reflected Georgia’s intermediate position between mainstream individualism and mainstream collectivism, once again underlining the relativity of the constructs and cautioning about the limits of their applicability to some cultures.

Age significantly correlated with cultural orientations confirming that younger generations in Georgia tend to be more individualistic. Nevertheless, the same younger generations still exhibited the key collectivistic trait: similar to the participants of Chinese and Italian studies (Germani et al., 2020; Wang et al., 2020), the majority worried about their family members contracting the virus and few worried about their own welfare.

Furthermore, while one third of the participants shared their households with three or more individuals, only 6% lived alone, and 28% lived with an elderly individual - all recognized features of collectivistic societies (Vandello and Cohen, 1999) – the scores of individualism and rational styles of coping significantly exceeded the scores of collectivism and affective styles of coping. In line with our results, another Eastern European study on the COVID-19 pandemic also showed that problem-focused coping exceeded emotion-focused coping (Szabó et al., 2020). The higher rates of individualism in our sample can be explained by the predominance of younger participants; in line with the previous study by Jamagidze et al. (2011), our results confirmed the growing individualism among younger generations of Georgia.

The higher rates of rational coping in our sample may be partly attributed to the fact that the study was conducted during the initial stage of pandemic when the epidemiological situation was very favorable (with daily rates of 0 deaths and an average number of new cases amounting to six only, World Health Organization), which made stressors fairly manageable. These results were further corroborated by the below average scores of both anxiety and COVID-19 worry among our sample. Repeating the survey by the end of 2020 (as planned), when the spread of infection reaches its peak (in mid-November, daily rates of deaths reached 40 and new cases exceeded 3000) may produce different results.

Thus, our results indicated that cultural characteristics and manageability of stressors need to be properly examined with respect to coping styles applied in response to the COVID-19 pandemic. In our case, specific cultural context stemming from a relatively intermediate position of Georgia (Eastern edge of Western world) between individualism and collectivism may partially be responsible for the following findings: COVID-19 worry, collectivism, and search for meaning in life – all predicted both rational and affective coping styles.

The findings pinpointed the variety of ways people perceive and react to a global threat and cope with the associated anxiety. They also expanded the cultural transactional theory of stress and coping by envisaging the concept of meaning in life through cultural lenses. On one hand, our results indicated that under collective crisis, such as a pandemic, everybody tends to be affected; yet, the specific reasons of why people become vulnerable may vary within the culture as well as across cultures, and identifying these reasons is crucial in defining proper intervention strategies. Airhihenbuwa et al. (2020) consider culture a central factor in ensuring an effective world-wide response to the global crises, stressing the importance of translating the unified global recommendations to the culture-relevant language. Thus, in a society like Georgia whose members are primarily worried about others’ wellbeing, support efforts should perhaps center on interdependence and on promoting ways for individuals to connect and care for each other. On the other hand, on a macro level, such a society will presumably better respond to the preventive slogans underlining responsibility for others (e.g., “protect your family,” “protect the elderly”) versus messages centered on self (e.g., “stay home,” “stay safe”).

Finally, the evidence generated by our sample indicated that some cultures may share characteristics of both individualistic and collectivistic societies and, therefore, display mixed representation of classic constructs. The current findings, thus, can contribute to cultural psychology research, inform practice and policy level decisions, and may be useful beyond the COVID-19 crisis.

## Limitations

The size of the sample, the broad geographic coverage, and the early post-outbreak study period can be considered as strengths of our study. Nevertheless, the research was not free of limitations. The main limitation was its bias stemming from convenience sampling that limits the generalizability of the findings. The sample mostly consisted of younger adults, primarily of the female gender. Besides, tech-savvy individuals were likely overrepresented. In addition, the level of distress probably influenced participants’ motivations to engage in the survey. Therefore, the extent of response bias in the data cannot be accurately estimated. The cross-sectional design of the current study also has its known drawbacks. Finally, the measure of coping styles used in the study is based on the Western understanding of coping and may overlook culturally congruent ways of coping.

## Conclusion

The stress caused by the pandemic created a natural milieu to examine links between anxiety, COVID-19 worry, and coping styles. We hypothesized that these links would be moderated by cultural orientations as well as meaning in life. Our hypotheses were supported in relation with some of these links. The main findings of our study suggested that cultural orientations and meaning in life predict rational and affective coping styles in a variety of ways, and moderate the links between anxiety and coping styles. Our findings concerning individualism/collectivism enriched and expanded the cultural transactional theory of stress and coping, while findings on meaning in life supported both culture-sensitive and generic approaches. The findings were explained within the complex context of the current outbreak and Georgia’s relatively intermediate position between clear-cut individualism and clear-cut collectivism and can be useful beyond the COVID-19 pandemic.

## Data Availability Statement

The raw data supporting the conclusions of this article will be made available by the authors, without undue reservation.

## Ethics Statement

The studies involving human participants were reviewed and approved by the Ilia State University Ethics Committee. Written informed consent for participation was not required for this study in accordance with the national legislation and the institutional requirements.

## Author Contributions

IS conceptualized the research, developed the study questionnaire, supported the data collection, performed the data analysis part and interpretation, and wrote the manuscript. NJ carried out the majority of data processing, analysis and interpretation, and contributed to the final manuscript. NC supported the study planning and data collection and contributed to the drafting, review, and editing of the manuscript. All authors approved the submitted version.

## Conflict of Interest

The authors declare that the research was conducted in the absence of any commercial or financial relationships that could be construed as a potential conflict of interest.
